# The mismatch repair system (*mutS* and *mutL*) in *Acinetobacter baylyi* ADP1

**DOI:** 10.1186/s12866-020-01729-3

**Published:** 2020-02-28

**Authors:** Hua Zhou, Linyue Zhang, Qingye Xu, Linghong Zhang, Yunsong Yu, Xiaoting Hua

**Affiliations:** 1 Department of Respiratory Diseases, the First Affiliated Hospital, Zhejiang University School of Medicine, Hangzhou, China; 2 Department of Infectious Diseases, Sir Run Run Shaw Hospital, Zhejiang University School of Medicine, Hangzhou, China; 3 Key Laboratory of Microbial Technology and Bioinformatics of Zhejiang Province, Hangzhou, China

**Keywords:** *Acinetobacter baylyi*, *mutS*, *mutL*, Mutation, Antibiotic resistance, Resistance evolution

## Abstract

**Background:**

*Acinetobacter baylyi* ADP1 is an ideal bacterial strain for high-throughput genetic analysis as the bacterium is naturally transformable. Thus, ADP1 can be used to investigate DNA mismatch repair, a mechanism for repairing mismatched bases. We used the *mutS* deletion mutant (XH439) and *mutL* deletion mutant (XH440), and constructed a *mutS mutL* double deletion mutant (XH441) to investigate the role of the mismatch repair system in *A. baylyi*.

**Results:**

We determined the survival rates after UV irradiation and measured the mutation frequencies, rates and spectra of wild-type ADP1 and *mutSL* mutant via rifampin resistance assay (Rif^R^ assay) and experimental evolution. In addition, transformation efficiencies of genomic DNA in ADP1 and its three mutants were determined. Lastly, the relative growth rates of the wild type strain, three constructed deletion mutants, as well as the rifampin resistant mutants obtained from Rif^R^ assays, were measured. All three mutants had higher survival rates after UV irradiation than wild type, especially the double deletion mutant. Three mutants showed higher mutation frequencies than ADP1 and favored transition mutations in Rif^R^ assay. All three mutants showed increased mutation rates in the experimental evolution. However, only XH439 and XH441 had higher mutation rates than the wild type strain in Rif^R^ assay. XH441 showed higher transformation efficiency than XH438 when donor DNA harbored transition mutations. All three mutants showed higher growth rates than wild-type, and these four strains displayed higher growth rates than almost all their *rpoB* mutants. The growth rate results showed different amino acid mutations in *rpoB* resulted in different extents of reduction in the fitness of rifampin resistant mutants. However, the fitness cost brought by the same mutation did not vary with strain background.

**Conclusions:**

We demonstrated that inactivation of both *mutS* and *mutL* increased the mutation rates and frequencies in *A. baylyi*, which would contribute to the evolution and acquirement of rifampicin resistance. The *mutS* deletion is also implicated in increased mutation rates and frequencies, suggesting that MutL may be activated even in the absence of *mutS*. The correlation between fitness cost and rifampin resistance mutations in *A. baylyi* is firstly established.

## Background

*Acinetobacter baylyi* ADP1 is a Gram-negative, non-pathogenic soil bacterium [1]. It is considered as an ideal organism for high-throughput genetic analysis, metabolic engineering and synthetic biology because it possesses a natural transformation system [2, 3]. Mutagenized DNA can be transformed into ADP1 to make sequence variations at specific sites in the genomic DNA [4]. Previously, the genome of *A. baylyi* ADP1 was sequenced [5], and a nearly complete collection of ADP1 mutants was constructed [2]. We used two mutants from this collection for further study.

DNA mismatch repair (MMR) is a DNA repair pathway focusing on repairing mismatched bases [6]. Mutations in DNA mismatch repair proteins (*mutS* and *mutL*) could confer hypermutator phenotypes and might facilitate the emergence of mutational antibiotic resistance in bacteria [7]. The MMR system was originally described in *Streptococcus pneumoniae* in 1962 [8, 9]. Currently, the best-characterized MMR systems are from *E. coli* and *Bacillus subtilis* [10]. The homologues of MutS exist in all prokaryotes, with the exception of *Actinobacteria*, *Mollicutes* and part of the *Archaea* [11]. Bacteria of the *Actinobacteria* Phylum and *Archaea* encode a non-canonical MMR system (NucS/endoMS) [12]. For *Mollicutes,* it was reported that the histone-like protein HU (Hup2) in *M. gallisepticum* might play it role in mismatch repair [13].

A previous study showed that strains lacking *mutS* exhibited increased spontaneous mutation frequencies in *A. baylyi*. Inactivating *mutS* also affected the transformation frequencies with divergent donor sequences with showing specificity for transition and frameshift mismatches in a marker replacement assay [14]. However, the influence of single *mutL* deletion and *mutS mutL* double deletion in ADP1 haven’t been studied yet. In this study, we constructed a *mutS mutL* double mutant (*ΔmutSΔmutL*: XH441), and we also adapted single deletion mutants (*ΔmutS*: XH439, *ΔmutL*: XH440) to provide a thorough understanding of the mismatch repair system in *A. baylyi*. We determined the survival rates after UV irradiation, and investigated the mutation frequencies, rates and spectrums of wild-type ADP1 (XH438), XH439*,* XH440, as well as XH441 via antibiotic rifampin resistance assay (Rif^R^ assay) and experimental evolution. In addition, transformation efficiencies of genomic DNA in ADP1 and its three mutants were determined. Lastly, the relative growth rates of the wild type strain, three constructed deletion mutants, as well as the antibiotic resistant mutants obtained from the Rif^R^ assay, were measured.

## Results

### Phenotypic characterization of the *mutS, mutL* single deletion mutants and the *mutS mutL* double deletion mutant

**Fig. 1:**
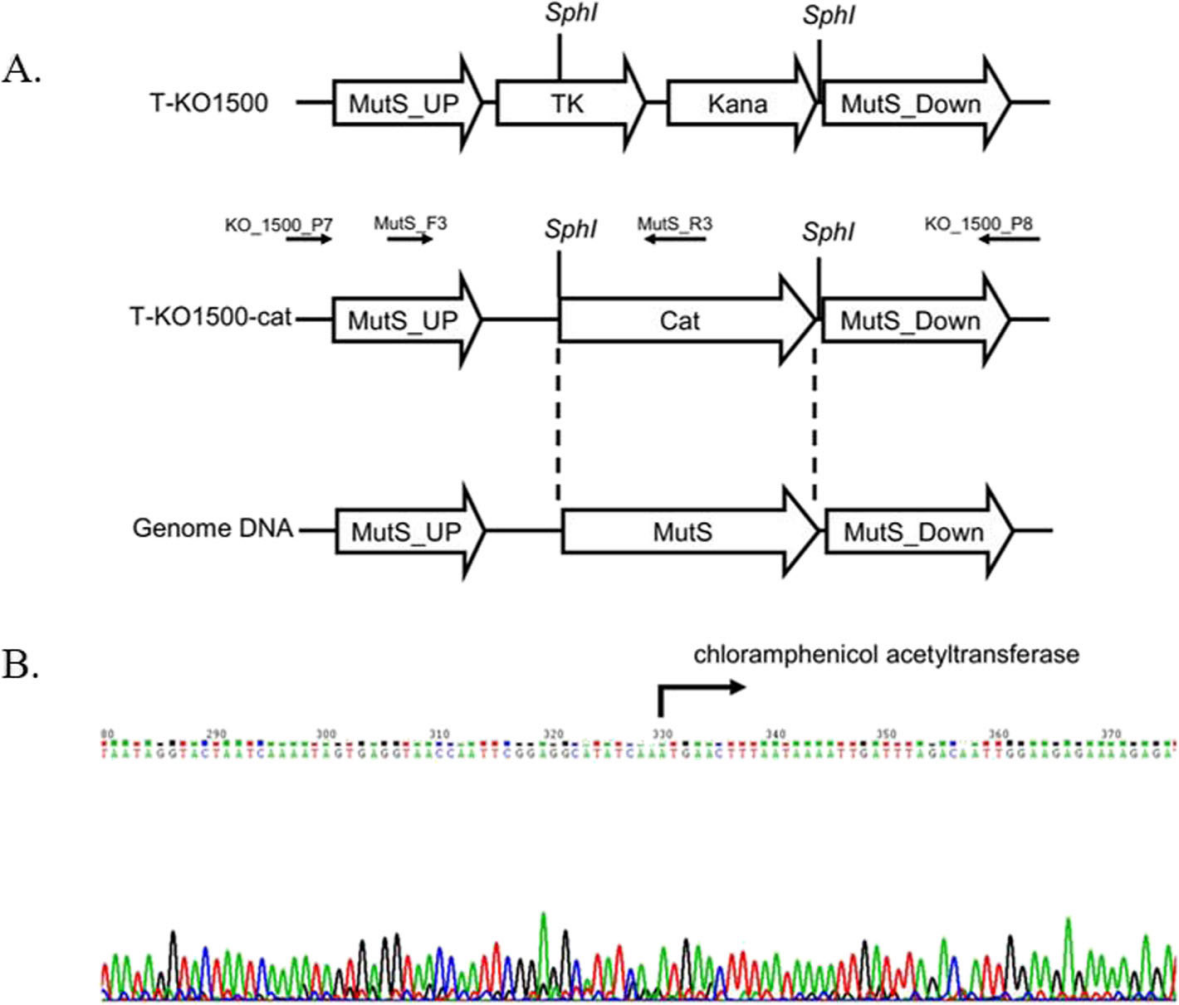
knockout of *mutS* based on *mutL* single deletion mutant. **a** Schematic representation of the targeting strategy for generating a *mutS mutL* double deletion mutant. **b** Fluorescent dye chemistry sequencing was performed using the same primers used for PCR. The arrow suggested the start site of cat gene

To identify the role of *mutS* and *mutL* in *A. baylyi*, we constructed a *mutS mutL* double deletion mutant (XH441) based on the *mutL* single deletion mutant XH440. A PCR fragment containing the *cat* gene which conferred chloramphenicol resistance and flanked by the regions surrounding the *mutS* gene, was introduced into XH440 by natural transformation (Fig. 1a). The *A. baylyi mutS mutL* double deletion mutant was obtained after selection on a chloramphenicol-containing plate. A specific PCR was used to confirm that the *mutS* gene was replaced by the cat gene, with subsequent sequencing of the fragment (Fig. 1b).

**Fig. 2:**
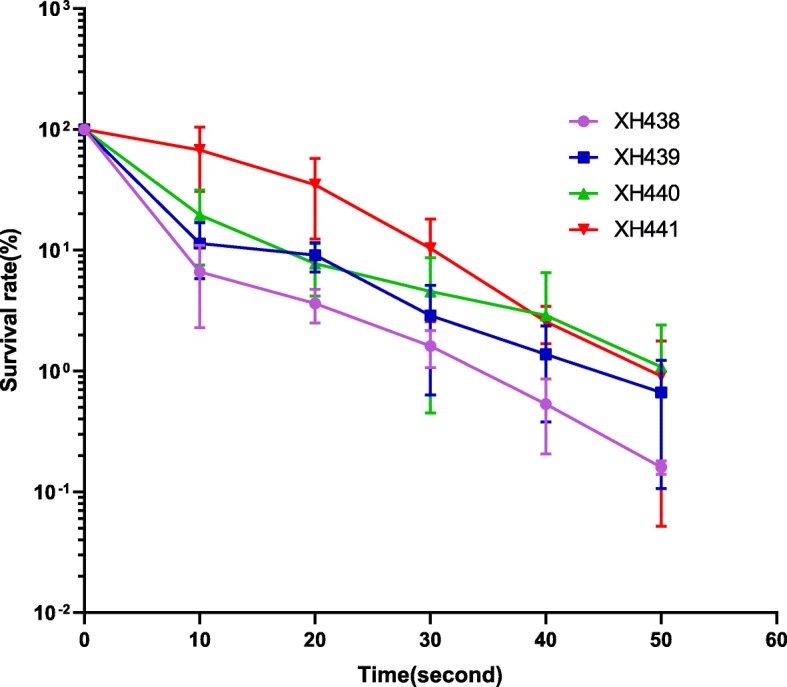
UV sensitivity of A. baylyi ADP1 (XH438) and various mutants. XH439 (*mutS*), XH440 (*mutL*), XH441 (mutS mutL). The data are means of three independent experiments, error bars represent standard deviation

After obtaining XH441, we determined the cell survival rates after UV irradiation of the single and double deletion mutants and compared the rates with that of wild-type *A. baylyi* ADP1. As shown in Fig. 2, all mutants showed higher survival rates than wild type, especially the double mutants. These results could implicate that an alternate repair pathway might be employed when the MMR system is non-functional.

### Mutation frequencies and mutation rates in Rif^R^ assay

The mutation frequencies and mutation rates of wild-type and mutant *A. baylyi* strains were determined in the Rif^R^ assay. Mutation frequencies of spontaneous occurrences of resistance towards rifampicin were determined and shown in Table 1. In the Rif^R^ assay, all three *mutS/mutL* single and double mutants showed significantly higher frequencies than wild type. Inactivation of *mutS* conferred a 4-fold increase (1.26 × 10^− 8^/3.02 × 10^− 9^) in the frequency of spontaneous rifampicin resistance compared to that of the wild type, whereas the mutation frequency of the *mutL* single mutant was 2-fold that of wild type (5.21 × 10^− 9^/3.02 × 10^− 9^). XH441 generated a mutation frequency 11-fold higher than that of wild type (3.42 × 10^− 8^/3.02 × 10^− 9^).

**Table 1:** Mutation frequencies and rates of XH438 (ADP1) and its deletion mutants in Rif^R^ assay

Strain	Genotype	Mutation frequency	Mutation rate
XH438	wild type	3.02 × 10^−9^	1.64 × 10^− 8^ (7.68 × 10^− 9^ – 2.98 × 10^− 8^)
XH439	*ΔmutS*	1.26 × 10^− 8^	3.02 × 10^− 8^ (1.88 × 10^− 9^ – 4.39 × 10^− 8^)
XH440	*ΔmutL*	5.21 × 10^− 9^	1.49 × 10^− 8^ (1.02 × 10^− 8^ – 2.06 × 10^− 8^)
XH441	*ΔmutSΔmutL*	3.42 × 10^− 8^	6.44 × 10^− 8^ (4.15 × 10^− 9^ – 9.09 × 10^− 8^)

For Rif^R^ assay, the mutation rate of the wild type strain ADP1 was estimated to be 1.6 × 10^− 8^ (95% confidence interval, 7.7 × 10^− 9^ to 3.0 × 10^− 8^) based on the number of derived Rif^R^ mutants. XH440 showed a similar mutation rate as the wild type (ADP1) (*p* = 0.802). Meanwhile, XH439 displayed an approximately2-fold elevated rate compared with the rate for ADP1, but these rates were not significantly different (*p* = 0.116). Only XH441 showed a 4-fold increased mutation rate compared with ADP1 (*p* < 0.01) (Table 1).

### Mutation rates based on experimental evolution with whole-genome sequencing

We performed laboratory evolution experiment with the wild type and its derivative deletion mutants (XH439, XH440, XH441) to determine their mutant rates under no antibiotic pressure. The mutation rates of four strains were shown in Table 2 and we obtained a similar increasing trend of mutation rates in the previous Rif^R^ assay except XH440 which showed a higher mutation rate in experimental evolution but a similar mutation rate in Rif^R^ assay compared with wild-type strain. The mutation rate of the wild type strain ADP1 was estimated as 2.99 × 10^− 9^ (95% confidence interval, 1.94 × 10^− 10^-4.16 × 10^− 9^) based on WGS analysis. All three mutants showed significantly higher point mutations rates than the wild type strain (5- to 9-fold).

**Table 2:** Mutation rates of XH438 (ADP1) and its deletion mutants in experimental evolution

Strain	Lines	transfer days	Generations	Ts	Tv	Indel	Point Mutation rate per nucleotide (μ_MA_)	95% confidence interval	Total Mutation rate per nucleotide (μ_MA_)	95% confidence interval
XH438	2	14	93	0	2	2	2.99 × 10^− 9^	1.94 × 10^−10^-4.16 × 10^− 9^	5.97 × 10^− 9^	2.20 × 10^− 9^-1.30 × 10^− 8^
XH439	1	14	93	8	1	4	2.69 × 10^− 8^	1.78 × 10^− 8^-3.92 × 10^− 8^	3.88 × 10^− 8^	3.50 × 10^− 8^-4.28 × 10^− 8^
XH440	1	14	93	5	0	6	1.49 × 10^−8^	8.39 × 10^− 9^-2.47 × 10^− 8^	3.28 × 10^− 8^	2.27 × 10^− 8^-4.63 × 10^− 8^
XH441	2	14	93	15	0	8	2.24 × 10^−8^	1.95 × 10^− 8^-2.55 × 10^− 8^	2.24 × 10^− 8^	1.37 × 10^− 8^-3.33 × 10^− 8^

*Ts* transition, *Tv* transversion, *Indel* insertion and deletion

### Mutational spectrums in Rif^R^ assay and experimental evolution

**Fig. 3:**
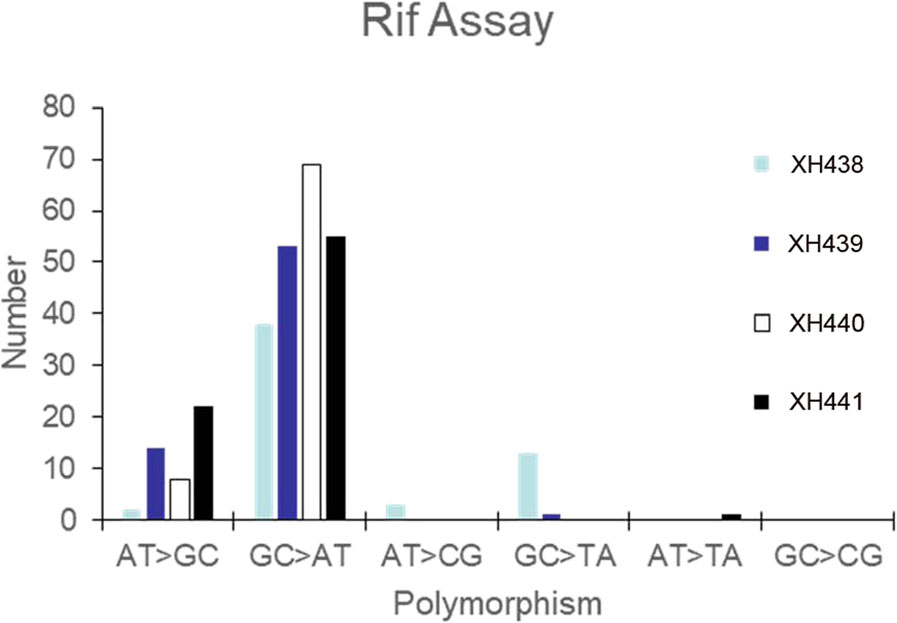
Comparison of percentage of transitions and transversions in *rpoB* in *A. baylyi* wild type and mutants in Rif^R^ assay

To understand the mechanism of mutation, we isolated rifampicin resistant mutants derived from wild-type, XH439, XH440 and XH441 in Rif^R^ assay and sequenced *rpoB* region which was related with Rif^R^. Table 3 showed 225 mutations leading to the Rif^R^ phenotype, including 58 mutations occurring in wild type, 77 mutations in XH439, 68 mutations in XH440 and 78 mutations in XH441. The proportion of transition in ADP1 was 71.4% (40 of 56). The transition mutations were favored in three mutants (Fig. 3). XH439 favored three transition sites at position 1619, 1562, and 1604. Only one prominent transition site, position 1619, remained in XH440. XH441 not only maintained one transition site at position 1619 but also showed two other transition sites at positions 1573 and 1574. Knockout of *mutL* or *mutS* increased the proportion of AT= > GC in transition mutations (Fig. 3).

**Table 3:** Distribution of mutations leading to Rif^R^ in *rpoB*

*A.baylyi* Site (bp)	Amino acid change	Base-pair change	XH438	XH439	XH440	XH441
1561	S521P	AT= > GC	2	0	1	6
1565	Q522R	AT= > GC	0	4	1	1
1574	D525G	AT= > GC	0	0	0	11
1604	H535R	AT= > GC	0	9	6	2
1625	L542S	AT= > GC	0	1	0	2
1562	S521F	CG= > TA	0	10	5	5
1573	D525N	CG= > TA	0	4	3	11
1592	S531F	CG= > TA	4	2	0	0
1603	H535Y	CG= > TA	6	1	5	4
1613	R538H	CG= > TA	0	1	1	3
1619	S540L	CG= > TA	11	28	48	27
1718	P573L	CG= > TA	17	7	7	5
1564	Q522K	CG= > AT	1	1	0	0
1603	H535N	CG= > AT	12	0	0	0
1741	I581F	AT= > TA	0	0	0	1
1604	H535P	AT= > CG	3	0	0	0
1569	12 bp insertion		2	0	0	0
Total			58	68	77	78

**Fig. 4:**
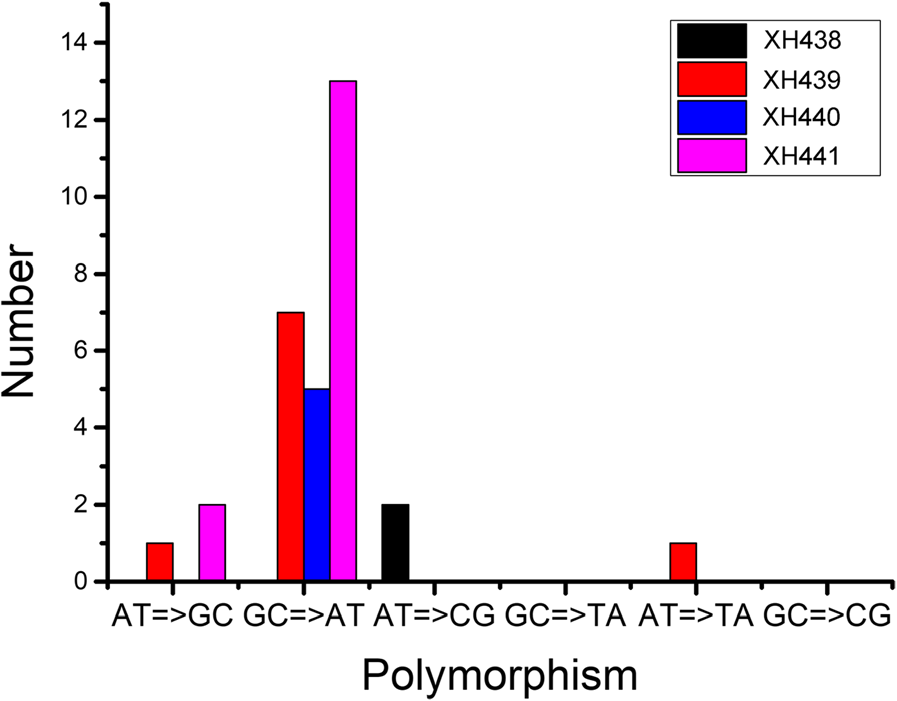
Comparison of percentage of transitions and transversions in the whole genome of *A. baylyi* wild type and mutants in experimental evolution

A similar pattern of mutations’ distribution was observed in experimental evolution. The sequence analysis of the final population evolved from wild type and its derivative deletion mutants (XH439, XH440, XH441) revealed a total of 4, 13, 11 and 23 mutations, respectively (Table 2, Table S1). Base substitutions were two-fold more common than insertions/deletions in XH439 and XH441 (Table 2), and transitions were more abundant than transversions in all mutants (Fig. 4). The mutations seemed randomly distributed throughout the *A. baylyi* genome (Table S1). During our study, we identified several genes as mutation hotspots. For example, all six strains harbored a mutation in intergenic region between ACIAD_RS11450 and ACIAD_RS11455 (divalent metal cation transporter/LysR family transcriptional regulator). In addition, mutations in ACIAD_RS05875 (universal stress protein) appeared in five strains (5/6). Further research is required to understand the roles of these mutations in *A. baylyi*.

### Effect of the *mutS* and *mutL* mutations on variations in marker replacement frequencies during transformation

**Fig. 5:**
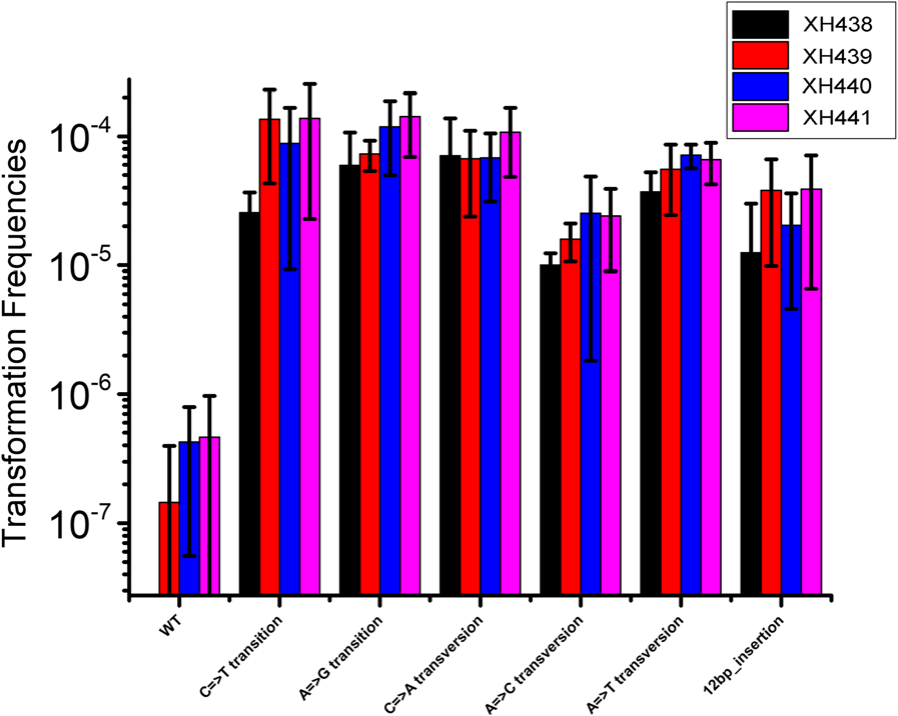
Transformation frequencies of donor DNA containing *rpoB* mutation. The transformation frequency was measured as transformants per CFU under normal growth condition

To detect the effect of *mutS* and *mutL* on the recognition of different mismatches in vitro, we transformed cells (wild-type, XH439, XH440, XH441) with genomic DNAs extracted from various spontaneous mutants obtained from Rif^R^ assay with a single point mutation (two transitions, three transversions) or 12 bp insertion in *rpoB* that resulted in Rif^R^ phenotype. The transformation frequencies of XH439 were 0.95- to 5.2-fold higher than those of the wild type (*p =* 0.33) (Fig. 5). XH440 also showed enhanced the transformation efficiency (0.97- to 3.5-fold) (*p =* 0.30). XH441 generated a higher transformation efficiency of DNA than single-gene deletion mutants (1.52 to 5.4-fold) (*p =* 0.02). No significant difference was detected in the interaction of receipt strain and donor DNA. Then, we divided the donor DNA into different mutation type, e.g. transition and transversion. XH441 showed a higher transformation efficiency than XH438 when donor DNA harbored transition mutations (*p* < 0.05).

### Effect of *rpoB* mutations on the relative growth rate

**Fig. 6:**
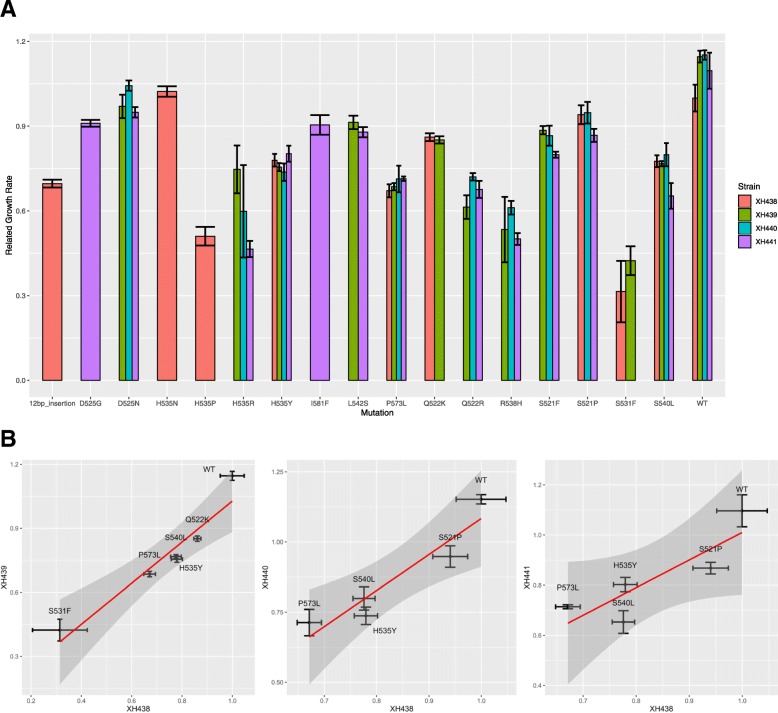
The relative growth rate of wild type and its derivative mutants, and their Rif^R^ mutants. **a** wild type and its derivative mutants, and their Rif^R^ mutants were inoculated overnight and diluted 1:100. 200 μl diluted culture was dispensed into quadruplicate wells of a Bioscreen C plate. The growth was monitored by measuring the OD_600_ value every 5 min for 16 h. Data represent the average of three technical replicates for three biologic samples; error bars indicated the SD. **b** the growth rate of wild type and its derivative mutants were plotted in pair. The correlation was evaluated using spearman correlation coefficient in R

To investigate whether the *rpoB* mutation affected the growth of *A. baylyi*, we determined the relative growth rate of wild type, its derivative mutant, and their Rif^R^ mutants. XH439, XH440 and XH441 all showed higher growth rates than wild-type XH438 (Fig. 6a). For Rif^R^ mutants, XH438 displayed higher growth rate than its *rpoB* mutants, except H535N and S521P. XH440 grew faster than its *rpoB* mutants, except D525N. XH439 and XH441 showed significant higher growth rate than their *rpoB* mutants (*p* < 0.05). The growth rates showed that different types of *rpoB* mutations conferred variable fitness costs to *A. baylyi*. Interestingly, the effect of the *rpoB* mutation on the growth rate was dependent on the mutation. We combined the growth rate data from different strains harboring different mutations, and compared them in pair. There was significant correlation between XH438 and its three deletion mutants (XH439, XH440, XH441) in growth rate (Fig. 6b). The spearman correlation coefficient between XH438 and its three deletion mutants are 0.94, 0.9 and 0.9, respectively.

## Discussion

In this study, we constructed a *mutS mutL* deletion mutant (XH441) and determined the mutation frequencies and rates in ADP1 (XH438), XH439, XH440 and XH441. XH439 (*ΔmutS*) showed a higher mutation frequency and mutation rate than XH440 (*ΔmutL*), which had a similar mutation rate and a slightly higher mutation frequency than the wild-type in Rif^R^ assay. Although mutL was quite important to MMR, as it was reported to act not only as a matchmaker, but also provide endonuclease activity for strand incision in *B. subtilis* [15]. According to out result, it seemed that mutL could not repair the mismatches without *mutS*, which resulted in higher mutation frequency and mutation rate in XH439 (*ΔmutS*). For the similar mutation rate and a slightly higher mutation frequency in XH440 (Δ*mutL*), we assumed that Rif^R^ mutants derived from XH440 might have no relationship with mismatch repair as MutS was reported to affect RecA-mediated DNA strand exchange independently of MutL both in *E. coli* [16] and *B. subtilis* [17]. But, in the experimental evolution, the mutation rate in XH440 was 5.5-fold higher than the wild-type. It might because that half of the mutations detected in XH440 using WGS were Indels which were not able to be detected in Rif^R^ assay (Table 2) (Table S1) as they did not occur on *rpoB*.

Mutations derived in a mismatch repair-deficient background are known to be predominantly GC= > AT or AT= > GC transitions [18, 19]. In our study, Rif^R^ assay and experimental evolution confirmed that the major mutations occurred on either *rpoB* or the whole genome were transition mutations. The increased proportion of AT= > GC transition mutations was more common in mutant strains. In Rif^R^ assay, we were able to detect mutations occurred on *rpoB* only. While the experimental evolution and the following WGS enabled us to detect additional types of mutations, e.g., short insertions or deletions and large deletions. The combination of experimental evolution and whole-genome sequencing provides an improved understanding of mutation formation in *A. baylyi*.

The MMR system was originally described in *S. pneumoniae* [8, 9]. At first, the proteins involved in the system were termed HexA and HexB. Previous study reported that the loss of *mutS* increased the transformation frequencies for the transition but did not affect the transformation frequencies of transversions in *A. baylyi* [14]. Our results showed that there are no significantly differences between the transformation frequencies of transition, transversion and insertion in four strains. Only XH441 showed a higher transformation efficiency than XH438 when donor DNA harbored transition mutations. The result confirmed that transition was favored in *mutSL* mutants.

Antibiotic resistance based on genetic mutations on chromosomes is often accompanied by fitness costs. Drug-resistant mutations in *rpoB* were mostly located in the 81-bp region of *rpoB* (rifampicin resistance determining region, RRDR). These *rpoB* mutations often come with fitness costs [20]. In this study, we reported the fitness cost of 14 types of *rpoB* base substitution and one type of 12-bp insertion. Most of *rpoB* mutation in four strains confirmed fitness cost. The growth rates showed that different types of *rpoB* mutations conferred variable fitness costs to *A. baylyi* and that there was also a large difference in the fitness of different missense mutations at the same amino acid position. The influence of *rpoB* mutations have been proved to be rather complex under different conditions in several studies. *rpoB* mutation in *Clostridium difficile* was associated with fitness cost in vitro and reduced virulence in vivo [21]. Mutations at position 522 or 540 in *rpoB* of *A. baumannii* were impaired in surface-associated motility and showed attenuated virulence [22]. The four different *rpoB* mutations in *E. coli* exhibited deleterious fitness costs under nutrient-rich conditions, but some *rpoB* alleles showed a remarkable fitness increase under phosphate limitation conditions [23]. In *S. aureus*, Rif^R^ mutants exhibited increased growth within biofilms [24]. The difference suggested that bacterial microenvironments should be considered before general conclusions on fitness cost are drawn [23]. To our knowledge, our study provides the first evaluation of the fitness cost associated with *A. baylyi* rifampin resistance in vitro.

## Conclusions

We demonstrated that inactivation of both *mutS* and *mutL* increased the mutation rates and frequencies in *A. baylyi*, which would contribute to the evolution and acquirement of rifampicin resistance. The *mutS* single deletion is also implicated in increased mutation rates and frequencies, suggesting that MutL may be activated even in the absence of *mutS*. The correlation between fitness cost and rifampin resistance mutations in *A. baylyi* is firstly established.

## Methods

### Bacterial strains, media and antibiotics

Restriction enzymes, T4 ligase, and Taq DNA polymerase were purchased from TaKaRa (Otsu, Shiga, Japan). All *A. baylyi* cultures (Table 4) were grown at 37 °C in LB broth and agar (Oxoid, Basingstoke, UK). Rifampin (Rif), kanamycin and chloramphenicol were purchased from Sangon (Shanghai, China) and dissolved in methanol.

**Table 4:** Bacterial strains and plasmids used in the study

Strain/ plasmid	Relevant characteristic(s)	Source
XH438	*A.baylyi* ADP1, Wild type	CEA
XH439	As ADP1 but *mutS*::kana	CEA
XH440	As ADP1 but *mutL*::kana	CEA
XH441	As KO2375 but *mutS*::cat	This study
T-KO1500	PCR fragment of flanking regions of kana which replaced *mutS* in XH440 cloned into T-vector, Amp^r^, Kana^r^	This study
T-KO1500-cat	PCR fragment of cat gene cloned into T-KO1500	This study

### Construction of *A. baylyi mutS mutL* mutant

*A. baylyi* ADP1, XH439, XH439 and XH440 were provided by the Commissariat à l’Energie Atomique/Direction des Sciences du Vivant [2]. We constructed a *mutS mutL* double mutant based on XH440. A DNA fragment was amplified from the XH439 mutant by primers KO_1500_P7 and KO_1500_P8 and ligated into the pMD 18-T vector as T-XH439. The cat gene was amplified from pTEX5500ts by cat_F2 and cat_R2, digested with *SphI*, and subcloned into T-XH439 as T-XH439-cat. The PCR fragment used to knockout *mutS* was amplified from T-XH439-cat and transformed into XH440, and the mutant was selected on a chloramphenicol-containing plate. The double mutant was verified by PCR and DNA sequencing.

### Survival rates characterization of *A. baylyi* and its mutants

UV irradiation experiment was conducted as described by Thoms and Wackernagel [25] with minor modifications. Bacteria overnight cultures in LB broth with a density of 10^8^ cells/ml were centrifuged and re-suspended in 1 × phosphate buffer (Senrui, China). Cells were irradiated at room temperature with a UV lamp in a biological safety cabinet (Thermo Scientific 1300 Series B2) at a distance of 60 cm. The cell suspension had a depth of less than 1 mm. Before and after irradiation, samples were withdrawn to estimate the survival rate of bacteria. Samples were plated on agar plates after appropriate dilution and colonies were counted after about 24 h incubation at 37 °C. The experiment was performed three times independently. Survival rates (%) were presented as the mean ± SD (standard deviation).

### Measurement of mutation frequency and mutation rate in Rif^R^ assay

For mutation frequency assays, overnight cultured cells were harvested, washed twice with phosphate-buffered saline (PBS) solution and re-suspended in PBS. Serial dilutions with PBS were plated onto LB plates containing 50 μg/mL Rif and incubated for 24 h before scoring. The total number of colony-forming units (CFU) was determined by plating serial dilutions on LB plates. The frequencies of mutations conferring resistance to Rif were determined by dividing the median number of mutants by the average number of cells [26].

For mutation rates, the maximum-likelihood estimator applying the newton. LD.plating function from the rSalvador package v1.7 for R was used to estimate the mutation rate (μ) to Rif^R^ in each strain, and statistical comparisons were performed by using the likelihood ratio test (LRT.LD.plating function from rSalvador) [27]. The colonies in the Rif^R^ LB plates were used for genomic DNA isolation, PCR, and Sanger sequencing.

### DNA isolation and *rpoB* sequencing of Rif^R^ mutants

Genomic DNA was isolated from the colonies in the Rif^R^ LB plates using a TianGen genome isolation kit (TianGen Biotech Company Ltd., Beijing, China). The primers ADP_rpoB_1S2 and ADP_rpoB_1A (Table 5) were used to amplify the DNA for sequencing. The PCR products were purified and sequenced by Biosune biological company (Hangzhou, China). The sequences and trace data were transferred to the SEQMAN program (DNASTAR), which was used for sequence assembly and SNP detection [26].

**Table 5:** primers used in the study

Primer name	Sequence(5′- > 3′)
KO_1500_P7	CCCATCTTTCTACAAGTAACGCTTAAACC
KO_1500_P8	CTAGACATTGGACAAAATAGCC
cat_F2	GCATGCCGTAAAATTTGTTTGATTTGTCC
cat_R2	GCATGCTTTCATTAGTCCATTACCTGGT
ADP_rpoB_1S2	TCGTTGCGGATACTTTGCGTGC
ADP_rpoB_1A	GCAAAGTTGGAACAGCCTGACG
MutS_F3	AAGCGAGATGTCTGTAGAAGTT
MutS_R3	GCTGTAATAATGGGTAGAAGGT

### Experimental evolution and measurement of mutation rate in *A. baylyi* ADP1 and its derivative mutants

A single colony of *A. baylyi* ADP1 and its derivative mutants were cultured in 2 mL of LB broth overnight at 37 °C. Then, the bacteria were serially passaged for 14 days. The final cultures were stored at − 80 °C. The genomic DNA was extracted and sequenced as previously described [28]. Briefly, the genomic DNA was extracted using a QIAamp DNA Mini Kit (Qiagen Valencia, CA). The genome was sequenced on an Illumina HiSeq platform (Illumina, San Diego, CA). Mutations in evolutionary strains were identified by Breseq [29]. The mutation rate was calculated via equation $\mu \mathrm{MA}=\frac{m}{\sum_{i=1}^n{N}_i\times {\mathrm{T}}_i}$, where *m* is the total number of mutations in all strains, *n* is the number of lines, *N*_*i*_ is the number of nucleotide sites, and *T* is the number of generations of bacteria during passage. Confidence intervals were calculated from a Poisson distribution using Poisson’s test in R [30].

### Natural transformation with genomic DNA with the *rpoB* mutation

For transformation with genomic DNA with the *rpoB* mutation, the genomic DNA were extracted from the known *rpoB* mutation isolates (including transition, transversion, and insertion). DNA was quantified by a NanoDrop ND-1000 spectrophotometer. Overnight cultured cells diluted 1:100 to fresh LB broth at 37 °C at 250 rpm for 2 h, and DNA was added to a final concentration of 400 ng/mL. After 3 h at 37 °C, cells were plated on LB plates, and LB plates contained 50 μg/mL rifampin. The plates were incubated for 24 h before scoring. The transformation frequencies were determined as the ratio of mutant bacteria on Rif^R^ plates to total viable bacteria on LB plates [31]. Three independent biological replicates were performed. ANOVA with TukeyHSD posthoc test was performed to identify differences between each strain when different donor DNA provided [32].

### Measurement of growth rate of ADP1 and derivative mutants

Three independent colonies per strain were grown in MH medium overnight and diluted to 1:100 in MH medium, and aliquots were placed into a flat-bottom 100-well plate in three replicates. Then, the plate was incubated at 37 °C, and the OD_600_ was detected every 5 min for 16 h using a Bioscreen C MBR machine (Oy Growth Curves Ab Ltd., Finland). The growth rate was estimated based on OD_600_ curves via BAT 2.0 [33]. The correlation of growth rate between different strains was evaluated using Spearman correlation coefficient in R [32]. The figures were plotted using ggplot2 [34].

## Supplementary information

**Additional file 1 **: **Table S1.** Predicted mutations in *A. baylyi* ADP1 from the WGS experiments.

## Data Availability

The whole-genome shotgun projects of the *A. baylyi* strains have been deposited at DDBJ/EMBL/GenBank under the accession numbers MPVT00000000-MPVY00000000. The versions described in this paper are versions MPVT00000000-MPVY00000000.

## Abbreviations

*A. baylyi*: *Acinetobacter baylyi*
CFU: Colony-forming units
MMR: Mismatch repair
PBS: Phosphate-buffered saline
Rif: Rifampin
Rif^R^: Rifampin resistance
UV: Ultraviolet
