# Supplementary material for: The mismatch repair system (mutS and mutL) in Acinetobacter baylyi ADP1
Source: BMC Microbiol. 2020 Feb 28;20:40. doi: 10.1186/s12866-020-01729-3 (PMC7048072; doi:10.1186/s12866-020-01729-3)
Supplement: Supplementary file 1 — Additional file 1 : Table S1. Predicted mutations in A. baylyi ADP1 from the WGS experiments. [file 12866_2020_1729_MOESM1_ESM.docx]

**Table S1** Predicted mutations from the WGS experiments

| **position** | **mutation** | **XH438-1*** | **XH438-2** | **XH439** | **XH440** | **XH441-1** | **XH441-2** | **annotation** | **gene** | **description** |
| --- | --- | --- | --- | --- | --- | --- | --- | --- | --- | --- |
| 98,603 | C→T |  |  |  |  | 100% | 100% | A100V (GCC→GTC) | *ACIAD_RS00470* → | glucose‑6‑phosphate isomerase |
| 178,459 | Δ1 bp |  |  | 100% | 100% | 100% | 100% | intergenic (+91/‑83) | *ACIAD_RS00825* → / → *ACIAD_RS00830* | hypothetical protein/ATP synthase subunit I |
| 242,874 | C→T |  |  | 100% |  |  |  | S272S (TCG→TCA) | *ACIAD_RS01140* ← | YifB family Mg chelatase‑like AAA ATPase |
| 387,161 | C→T |  |  |  |  | 100% | 100% | S132S (AGC→AGT) | *ACIAD_RS01825* → | polyphosphate‑‑AMP phosphotransferase |
| 439,244 | C→T |  |  | 100% |  |  |  | G61D (GGT→GAT) | *ACIAD_RS02030* ← | Rne/Rng family ribonuclease |
| 488,558 | C→T |  |  |  |  | 100% |  | A31V (GCA→GTA) | *ACIAD_RS02245* → | ATP‑dependent helicase |
| 574,644 | Δ1 bp |  |  | 100% |  |  |  | coding (208/435 nt) | *ACIAD_RS02665* → | hypothetical protein |
| 653,286 | G→A |  |  |  | 100% |  |  | intergenic (+146/‑23) | *ACIAD_RS03040* → / → *ACIAD_RS03045* | RNA polymerase factor sigma‑54/ribosome‑associated translation inhibitor RaiA |
| 1,073,998 | C→T |  |  |  | 100% |  |  | R74* (CGA→TGA) | *ACIAD_RS16735* → | DUF1989 domain‑containing protein |
| 1,236,402 | C→T |  |  |  |  | 100% | 100% | A87T (GCA→ACA) | *ACIAD_RS05690* ← | methyltransferase domain‑containing protein |
| 1,278,175 | +T |  |  | 100% |  |  |  | coding (348/888 nt) | *ACIAD_RS05875* → | universal stress protein |
| 1,278,176 | Δ1 bp | 100% |  |  |  | 100% | 100% | coding (349/888 nt) | *ACIAD_RS05875* → | universal stress protein |
| 1,278,657 | Δ1 bp |  | 100% |  |  |  |  | coding (830/888 nt) | *ACIAD_RS05875* → | universal stress protein |
| 1,358,156 | +A |  |  |  | 100% |  |  | coding (55/2277 nt) | *ACIAD_RS06285* → | ATP‑dependent Clp protease ATP‑binding subunit ClpA |
| 1,363,363 | +T |  |  |  |  | 100% | 100% | coding (121/657 nt) | *ACIAD_RS06305* ← | TetR/AcrR family transcriptional regulator |
| 1,401,404 | Δ1 bp |  |  |  | 100% |  |  | coding (108/273 nt) | *ACIAD_RS06500* → | HU family DNA‑binding protein |
| 1,498,517 | T→C |  |  |  |  | 100% | 100% | intergenic (‑85/‑143) | *ACIAD_RS06920* ← / → *ACIAD_RS06925* | DNA mismatch repair protein MutS/hypothetical protein |
| 1,670,292 | G→A |  |  |  | 100% |  |  | Q53* (CAG→TAG) | *ACIAD_RS07670* ← | 1‑acyl‑sn‑glycerol‑3‑phosphate acyltransferase |
| 1,687,712 | C→T |  |  |  | 100% |  |  | S68L (TCG→TTG) | *ACIAD_RS07755* → | CoA transferase |
| 1,929,169 | Δ931 bp |  |  |  | 100% |  |  |  | *[ACIAD_RS08925]*–*[ACIAD_RS08930]* | *[ACIAD_RS08925], [ACIAD_RS08930]* |
| 2,009,245 | T→C |  |  | 100% |  |  |  | T313T (ACT→ACC) | *ACIAD_RS09285* → | alpha/beta hydrolase |
| 2,087,703 | G→A |  |  | 100% |  |  |  | W543* (TGG→TAG) | *ACIAD_RS09635* → | OFA family MFS transporter |
| 2,099,940 | +T |  |  | 100% |  |  |  | coding (313/468 nt) | *ACIAD_RS09685* → | BLUF domain‑containing protein |
| 2,243,198 | G→A |  |  | 100% |  |  |  | intergenic (+654/+12) | *ACIAD_RS10405* → / ← *ACIAD_RS10410* | OmpW family protein/Si‑specific NAD(P)(+) transhydrogenase |
| 2,320,433 | G→A |  |  |  | 100% |  |  | P27L (CCG→CTG) | *ACIAD_RS10800* ← | DNA polymerase III subunit delta' |
| 2,468,889 | G→A |  |  | 100% |  |  |  | R213H (CGC→CAC) | *ACIAD_RS11410* → | CoA transferase subunit A |
| 2,478,738 | A→T |  |  | 100% |  |  |  | intergenic (‑98/+265) | *ACIAD_RS11450* ← / ← *ACIAD_RS11455* | divalent metal cation transporter/LysR family transcriptional regulator |
| 2,478,740 | Δ4 bp |  |  |  | 100% | 100% | 100% | intergenic (‑100/+260) | *ACIAD_RS11450* ← / ← *ACIAD_RS11455* | divalent metal cation transporter/LysR family transcriptional regulator |
| 2,478,767 | T→G | 100% | 100% |  |  |  |  | intergenic (‑127/+236) | *ACIAD_RS11450* ← / ← *ACIAD_RS11455* | divalent metal cation transporter/LysR family transcriptional regulator |
| 2,617,655 | G→A |  |  |  |  | 100% | 100% | G28R (GGA→AGA) | *ACIAD_RS12110* → | DNA repair protein RadA |
| 2,713,687 | G→A |  |  |  |  | 100% | 100% | R619W (CGG→TGG) | *ACIAD_RS12550* ← | peptide synthetase |
| 2,958,205 | G→A |  |  | 100% |  |  |  | V253V (GTC→GTT) | *ACIAD_RS13690* ← | DUF2339 domain‑containing protein |
| 3,061,520 | G→A |  |  | 100% |  |  |  | L495L (CTG→CTA) | *ACIAD_RS14165* → | FAD‑dependent 5‑carboxymethylaminomethyl‑2‑thiouridine(34) oxidoreductase MnmC |
| 3,135,968 | +A |  |  |  | 100% |  |  | coding (115/732 nt) | *ACIAD_RS14605* ← | type 1 glutamine amidotransferase |
| 3,250,534 | C→T |  |  |  |  | 100% | 100% | G339D (GGC→GAC) | *ACIAD_RS15135* ← | hypothetical protein |

*XH438 and XH441 harbored two bioreplicate, while XH439 and XH440 only have one bioreplicate.
